# Molecular basis of inhibition of acid sensing ion channel 1A by diminazene

**DOI:** 10.1371/journal.pone.0196894

**Published:** 2018-05-21

**Authors:** Aram J. Krauson, James G. Rooney, Marcelo D. Carattino

**Affiliations:** 1 Renal-Electrolyte Division, Department of Medicine, University of Pittsburgh, Pittsburgh, Pennsylvania, United States of America; 2 Department of Cell Biology, University of Pittsburgh, Pittsburgh, Pennsylvania, United States of America; Ohio State University, UNITED STATES

## Abstract

Acid-sensing ion channels (ASICs) are trimeric proton-gated cation permeable ion channels expressed primarily in neurons. Here we employed site-directed mutagenesis and electrophysiology to investigate the mechanism of inhibition of ASIC1a by diminazene. This compound inhibits mouse ASIC1a with a half-maximal inhibitory concentration (IC_50_) of 2.4 μM. At first, we examined whether neutralizing mutations of Glu79 and Glu416 alter diminazene block. These residues form a hexagonal array in the lower palm domain that was previously shown to contribute to pore opening in response to extracellular acidification. Significantly, single Gln substitutions at positions 79 and 416 in ASIC1a reduced diminazene apparent affinity by 6–7 fold. This result suggests that diminazene inhibits ASIC1a in part by limiting conformational rearrangement in the lower palm domain. Because diminazene is charged at physiological pHs, we assessed whether it inhibits ASIC1a by blocking the ion channel pore. Consistent with the notion that diminazene binds to a site within the membrane electric field, diminazene block showed a strong dependence with the membrane potential. Moreover, a Gly to Ala mutation at position 438, in the ion conduction pathway of ASIC1a, increased diminazene IC_50_ by one order of magnitude and eliminated the voltage dependence of block. Taken together, our results indicate that the inhibition of ASIC1a by diminazene involves both allosteric modulation and blocking of ion flow through the conduction pathway. Our findings provide a foundation for the development of more selective and potent ASIC pore blockers.

## Introduction

Acid-sensing ion channels (ASICs) are proton-gated cation permeable ion channels that are expressed in neurons of the peripheral and central nervous system. These proteins are members of the epithelial sodium channel/degenerin (ENaC/deg) family, which encompasses ion channels expressed in animals from nematodes to mammals. Four genes that encode for six ASIC subunits and splice variants have been identified in mammals (ASIC1a, ASIC1b, ASIC2a, ASIC2b, ASIC3, and ASIC4) [1–10]. ASIC subunits assemble to form homo- and hetero-trimers with distinctive biophysical properties including, agonist affinity, single channel conductance, rate of desensitization and of recovery after desensitization, and cation selectivity [11, 12]. When expressed in heterologous expression systems, only ASIC1a, ASIC1b and ASIC3 respond to changes in extracellular pH within the physiological range [3, 12]. ASIC1a is primarily expressed in the cell body, dendrites and dendritic spines of neurons in the central nervous system [13, 14]. Studies conducted with ASIC1a null animals indicate that the expression of this channel in the central nervous system is necessary for proper development of cognitive functions such as learning and memory [14, 15]. Substantial evidence favors the notion that the activation of ASIC1a has a detrimental contribution in various animal model of neurological diseases. For instance, genetic ablation and pharmacological blockade of ASIC1a have been shown beneficial in animal models of ischemic stroke [16], multiple sclerosis [17], Parkinson’s disease [18], Huntington’s disease [19], migraine [20], and glioblastoma [21–24]. For ASIC1b, little is known about its expression and physiological roles. Lastly, ASIC3 is primarily expressed in the soma and terminals of sensory neurons in the peripheral nervous system where it contributes to nociception and mechanosensation [25, 26].

While therapies that target ASICs can potentially be employed to treat several neurological diseases, up to now a limited number of ASIC inhibitors have been identified. The most selective and potent inhibitors of ASICs are polypeptides found in venoms including psalmotoxin (PcTx1), a toxin isolated from the tarantula *Psalmopoeus cambridgei* that desensitizes ASIC1a homomers and ASIC1a-ASIC2b heteromers [27–29]; APETX2, a toxin isolated from the sea anemone *Anthopleura elegantissima* that inhibits ASIC3 and ASIC3-containing heteromers [30]; and mambalgin-1, a toxin isolated from the Black mamba that inhibits ASIC1a and ASIC1b homomers, and ASIC1a-ASIC2a, ASIC1a-ASIC2b and ASIC1a-ASIC1b heteromers [31]. Several small molecules were reported to inhibit the activity of all ASIC subtypes including, some non-steroidal anti-inflammatory drugs (flurbiprofen, ibuprofen, aspirin, salicylic acid and diclofenac) [32, 33], amiloride [11], 5-(N-Ethyl-N-isopropyl)amiloride [10, 34], A-317567 [35, 36], nafamostat [37], and a group of diarylamidines (diminazene, 4’,6-diamidino-2-phenylindole (DAPI), hydroxysitlbamidine and pentamidine) [38]. Among the small inhibitors of ASICs, diminazene showed high potency with similar efficacy for ASIC1a, ASIC1b, ASIC2a and ASIC3 homomers [38].

In this manuscript, we combined site-directed mutagenesis and electrophysiology to define the mechanism of inhibition of ASIC1a by diminazene. We chose to examine the mechanism of inhibition of ASIC1a by diminazene because the high potency and specificity of this compound toward ASIC family members. Consistent with combined allosteric and pore blocking mechanisms of action, site-directed mutagenesis studies mapped diminazene binding sites to the lower palm region and the permeation pathway of the channel. The findings reported here provide a molecular framework for the development of more selective and potent ASIC pore blockers.

## Materials and methods

### Molecular biology and oocyte expression

Murine ASIC1a in a pSP64 Poly(A) (Promega) was mutated using Quickchange XL site-directed mutagenesis kit (Agilent Technologies). All ASIC1a constructs carried a C70L mutation. All constructs were confirmed by direct sequencing. cDNAs were *in vitro* transcribed with SP6 mMessage mMachine (Applied Biosystems) according to the manufacturer’s instructions. Oocytes in stages 5–6 were harvested from adult female Xenopus laevis (NASCO) in accordance with a protocol approved by the University of Pittsburgh Institutional Animal Care and Use Committee. Oocytes were injected with 0.2–6 ng of cRNA coding for ASIC wild type and mutant channels and maintained at 18°C in modified Barth's solution (MBS) containing (mM) 88 NaCl, 1 KCl, 2.4 NaHCO_3_, 15 HEPES, 0.3 Ca(NO_3_)_2_, 0.41 CaCl_2_, 0.82 MgSO_4_, 10 μg/ml sodium penicillin, 10 μg/ml streptomycin sulfate, 100 μg/ml gentamycin sulfate, pH 7.4.

### Electrophysiology

Experiments were conducted 24–48 h after injection at room temperature (20–25°C) as previously described [39–42]. Oocytes were placed in a recording chamber with a volume of approximately 20 μl (Automate Scientific) and impaled with glass electrodes filled with 3 M KCl. The resistance of the glass electrodes was 0.2–2 megaohms. Fluid was delivered to the chamber at a rate of 8–10 ml/min using a gravity-feed perfusion system (Automate Scientific). Two-electrode voltage clamp was performed with a TEV-200A amplifier (Dagan). Data were captured with a Digidata 1440A acquisition system using pClamp 10 (Molecular Devices). Currents were measured at a holding potential of -60 mV, unless otherwise indicated. The recording solution contained (in mM) 110 NaCl, 2 KCl, 1 CaCl_2_, 10 Hepes, pH 8.0. Acidic test solutions of pH 5.0–6.5 were buffered with MES.

### Spectroscopic analysis of diminazene

Diminazene solutions (20 μM) were prepared in 20 mM sodium phosphate buffered at pH 1–13. Spectrums were acquired with in an UV-1800 spectrophotometer (Shimadzu) with quartz cuvettes. The spectrophotometer was set to record a spectrum between wavelengths of 240–550 nm. Baseline was corrected with sodium phosphate solutions of corresponding pH. Data were analyzed with UVProbe 2.33 (Shimadzu).

### Data and statistical analysis

Data are expressed as means ± SEM (n), where n equals the number of independent experiments analyzed. Kruskal-Wallis test followed by Dunn’s multiple comparisons test was employed to compare time constant of desensitization between groups, and p < 0.05 was considered statistically significant. The half-maximal inhibitory concentration (IC_50_) for diminazene is expressed as the mean with a 95% confidence interval (CI). IC_50_s were calculated from normalized currents plotted as a function of the diminazene concentration according to the following equation, *y* = *max* + (*basal* − *max*)/(1 + 10^(*X*−*Log IC*50)^), where *y* is the response variable, *x* is the concentration of diminazene, and IC_50_ is the concentration of diminazene that inhibits pH-evoked current halfway between the basal response (*basal*) and complete inhibition (*max*). To assess the impact of mutations on diminazene IC_50_, 95% confidence intervals for the means were compared. The means of two groups were considered statistically significant (at the 0.05 level of significance) when the 95% confidence intervals for the means did not overlap. To examine the voltage dependence of diminazene inhibition, current-voltage (I-V) relationships were generated by plotting the currents evoked by 500 msec voltage ramps between -120 mV and -40 mV. I-V relationships were calculated by subtracting the currents measured at pH 8.0 from those measured at a pH of 6.5. Fitting of dose-response relationships and statistical comparisons were performed with GraphPad 5.03 (GraphPad Software).

## Results

**Fig 1:**
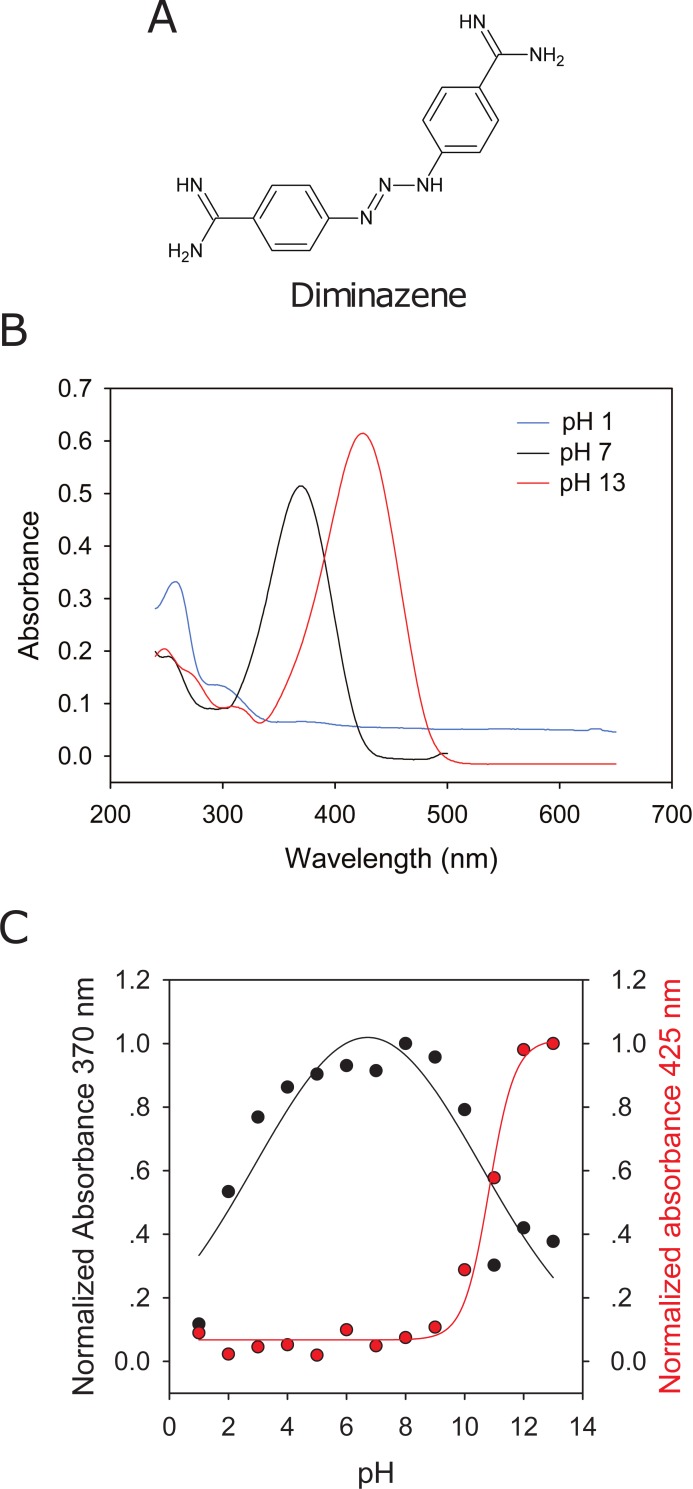
Diminazene is protonated at physiological pHs. (A) Chemical structure of diminazene. (B) Representative absorbance spectrums of diminazene in sodium phosphate solutions of pH 1, 7 and 13. Buffered solutions prepared as described in Materials and Methods contained 20 μM diminazene. The wavelength of maximum absorbance for diminazene at pH 7 and 13 were 370 nm and 425 nm, respectively. (C) Diminazene absorption as function of pH. Absorbance at 370 nm was normalized to the absorbance value of the diminazene solution at pH 8 (black dots, left y-axis). Absorbance at 425 nm was normalized to the absorbance value of the diminazene solution of pH 13 (red dots, right y-axis). Data are representative of two experiments.

Diminazene is a trypanocidal agent that belongs to the phenylhydrazines and contains two amidine groups attached to benzene groups (Fig 1A). The nitrogen atoms in the hydrazine and amidine groups of diminazene can undergo protonation as a function of the pH. To determine whether diminazene is protonated at physiological pHs, we collected absorption spectra for diminazene in phosphate buffered solutions of pH 1–13. Three main peaks were observed in the absorption spectra, a peak with a wavelength maxima at 425 nm noticeable in solutions of pH 10–13, a peak with a wavelength maxima at 370 nm present in solutions of pH 2–10, and a peak with a wavelength maxima at 258 nm observed in solutions of pH 1 (Fig 1B). We assume that at pH 13 diminazene exists in a non-protonated state. The changes in absorbance at 425 nm and 370 nm are consistent with the protonation of the amidine and hydrazine groups. These results indicate that in the range of pH used for our experiments (pH 5–8) diminazene is protonated.

**Fig 2:**
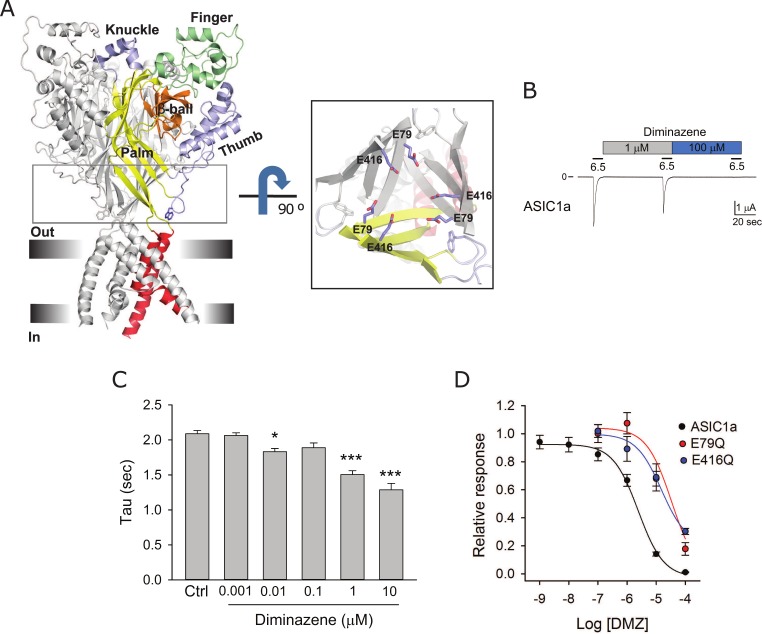
Neutralization of Glu79 and Glu416 increases diminazene half-maximal inhibitory concentration. (A) Cartoon representation of chicken ASIC1 (cASIC1) in the desensitized state (PBD 4NYK). Inset, Close up view of residues Glu79 and Glu416 in the palm domain. (B) Representative tracing showing the inhibitory effect of diminazene on ASIC1a. Whole-cell currents were evoked by a change in extracellular pH from 8.0 to 6.5. (C) Time constant of desensitization (tau) for ASIC1a in the absence and presence of increasing concentrations of diminazene. Statistically significant differences in time constants of desensitization of ASIC1a before (control) and after diminazene are indicated as * p<0.05 and ** p<0.001 (n = 11–39, Kruskal-Wallis test followed by Dunn’s multiple comparisons test). (D) Neutralization of Glu79 and Glu416 in ASIC1a increases diminazene IC_50_. Whole-cell currents were evoked by a change in extracellular pH from 8.0 to solutions of lower pH. pH of activation was 6.5 for wild type ASIC1a and 5 for E79Q and E416Q. The relative response represents the ratio of the pH-elicited peak current after diminazene to the pH-elicited peak current before diminazene. Diminazene IC_50_ for ASIC1a was 2.42 μM (CI 1.56–3.75 μM, n = 10–14), for E79Q was 18.42 μM (CI 8.78–49.75 μM, n = 9–10) and for E416Q was 13.89 μM (CI 5.44–38.23 μM, n = 9–13).

ASIC subunits contain two transmembrane spanning domains connected by a large extracellular loop with intracellular N- and C- termini. The transmembrane (TM) domains assemble to form the pore of the channel (Fig 2). Residues from the second transmembrane (TM2) line the permeation pathway and residues from the first transmembrane (TM1) domain reside in the periphery and interface with the lipid bilayer. The extracellular region encompasses about 400 residues and it is organized in well-defined domains termed finger, palm, thumb, beta-ball, and knuckle (Fig 2A). The structure of chicken ASIC1 (cASIC1) in the desensitized-like state reveals the presence of a hexagonal array of acidic residues formed by Glu79 and Glu416 in the lower palm domain (Fig 2A inset). We previously showed that the protonation of these residues facilitates pore opening in response to extracellular acidification [41]. To determine whether diminazene block involves the binding to the array of acidic residues in the palm domain, we generated diminazene dose-response curves for ASIC1a bearing single neutralizing mutations (Glu to Gln) at positions 79 and 416. ASIC1a was activated by a drop in extracellular pH from 8 to 6.5, which produces near half-maximal activation [41, 42]. Glu to Gln mutations at positions 79 and 416 in ASIC1a reduce proton apparent affinity and produce biphasic dose-response activation curves [41, 42]. Thus, for these mutants we used a pH of activation of 5, which produces 50% of maximal activation [42]. Although at high concentrations diminazene accelerated the desensitization of ASIC1a (Fig 2C), the observed changes were relatively modest. Thus, to assess the impact of mutations on diminazene inhibition we measured peak currents. Consistent with previous studies [38], diminazene IC_50_ for ASIC1a was 2.42 μM (CI 1.56 to 3.75 μM) (Fig 2D). Single Glu to Gln mutations at positions 79 and 416 in ASIC1a shifted diminazene apparent affinity to 18.42 μM (CI 8.78 to 49.75 μM) and 13.89 μM (CI 5.44 to 38.23 μM), respectively (Fig 2D). Note that the maximal concentration of diminazene that we can use in our experiments without affecting oocyte integrity is 100 μM. Then, IC_50_ values estimated for E79Q and E416Q are approximations. We were not able to assess whether mutations at both positions have an additive effect on diminazene block because E79Q-E416Q channels are electrically silent [41]. Together, these results suggest that Glu79 and Glu416 are part of a diminazene coordination site in the lower palm domain.

**Fig 3:**
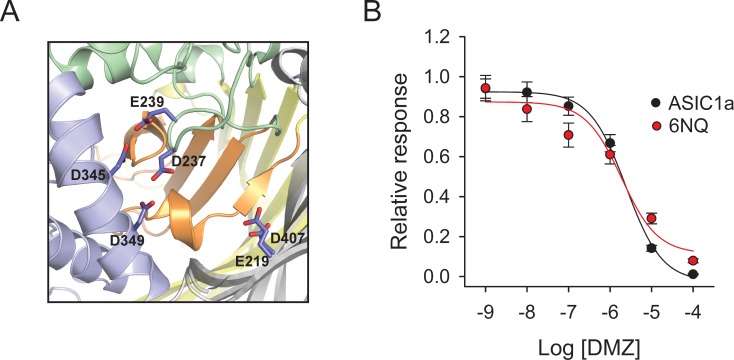
Neutral substitutions at the acidic pocket do not alter diminazene block. (A) Cartoon representation of the acidic pocket of cASIC1 (PBD 4NYK). (B) Diminazene dose response curves for wild type ASIC1a and the 6NQ (E219Q/D237N/E238Q/D345N/D349N/D407N) mutant. Whole-cell currents were evoked by a change in extracellular pH from 8.0 to 6.5. The relative response represents the ratio of the pH-elicited peak current after diminazene to the pH-elicited peak current before diminazene. Diminazene IC_50_ for ASIC1a was 2.42 μM (CI 1.56–3.75 μM, n = 10–14) and for the 6NQ mutant was 2.03 μM (CI 0.88–5.31 μM, n = 10–21).

Analysis of the electrostatic potential of chicken ASIC1 (cASIC1) mapped on the solvent-accessible surface disclosed an “acidic pocket” formed by residues at the interface between the thumb domain, finger, beta-ball, together with residues from the palm domain on an adjacent subunit (Fig 3A). Residues in the acid pocket serve as binding sites for psalmotoxin [43]. To determine whether diminazene interacts with residues in the acidic pocket, we introduced amino acids with polar uncharged side chains at positions 219, 237, 238, 345, 349 and 407 (6N/Q) in ASIC1a and generated a diminazene dose-response curve for this mutant (Fig 3B). As shown in Fig 3B, the introduction of neutral mutations in the acidic pocket did not change diminazene apparent affinity.

**Fig 4:**
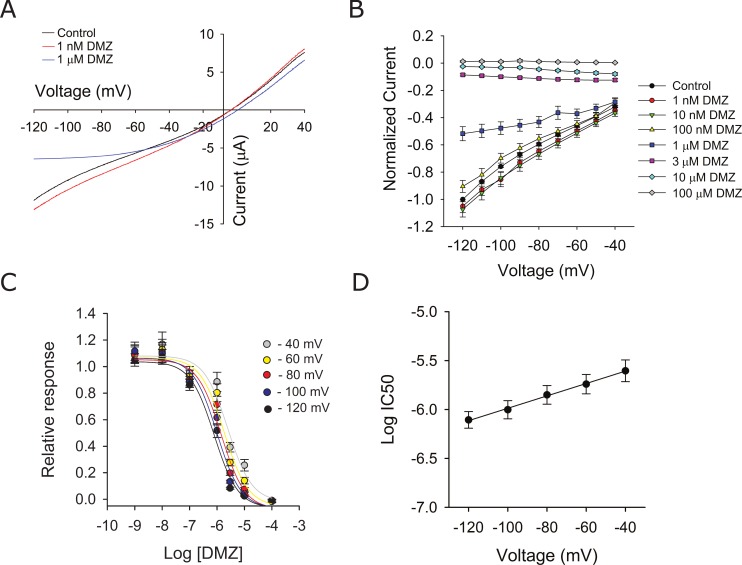
Voltage-dependent inhibition of ASIC1a by diminazene. (A) Representative ramp tracings from an oocyte expressing ASIC1a before and after diminazene. Ramps from +60 to -140 mV with a duration of 500 msec were generated in the absence and presence of diminazene (1 nM and 1 μM). ASIC currents were evoked by changing the extracellular pH from 8 to 6.5. Note that the inhibition of ASIC1a by 1 μM diminazene displays strong voltage dependence. (B) Normalized proton-evoked currents from oocytes expressing ASIC1a in the absence and presence of diminazene. Proton-evoked currents were elicited as indicated above in B. Proton-evoked currents measured in the presence of diminazene were normalized to the proton-evoked current measured at -120 mV before diminazene (n = 7–22). (C) Diminazene IC_50_ at each voltage was calculated from dose-response curves. The calculated diminazene IC_50_ was 2.48 μM (CI 1.48–4.04 μM, n = 7–22) at—40 mV, 1.82 μM (CI 1.16–2.75 μM, n = 7–22) at—60 mV, 1.41 μM (CI 0.91–2.10 μM, n = 7–22) at -80 mV, 0.99 μM (CI 0.63–1.49 μM, n = 7–22) at -100 mV, and 0.78 μM (CI 0.52–1.13 μM, n = 7–22) at -120 mV. (D) Diminazene IC_50_ shows a strong dependence with the membrane potential. Log IC_50_ values (C) were plotted as a function of the membrane potential. Data represent log IC_50_ ± SEM. Data were fitted by linear regression (slope 0.0063 ± 0.0002, r^2^ = 0.997, p<0.0001).

A hallmark of pore blockers is that their apparent affinity varies with membrane potential. To determine whether diminazene block is voltage dependent, we generated ramps from +60 to -140 mV with a duration of 500 msec in the absence and presence of diminazene. ASIC1a currents were evoked by changing the extracellular pH from 8 to 6.5. As shown in Fig 4A, the effects of voltage on diminazene block were marked at negative membrane potentials. Consequently, we plotted normalized proton-evoked currents between membrane potentials of -40 mV and -120 mV. I-V relationships were calculated by subtracting the currents measured at pH 8.0 from those measured at pH 6.5. For each experiment, proton-evoked currents at a given membrane potential were normalized to the current evoked by extracellular acidification at a membrane potential of -120 mV in the absence of diminazene (Fig 4B). Diminazene IC_50_ for each voltage was calculated from dose-response curves (Fig 4C). As shown in Fig 4D, diminazene IC_50_ shows a strong dependence with the membrane potential. These results are consistent with the notion that diminazene binds to a site within the membrane electric field to inhibit ion permeation through the pore of ASIC1a.

**Fig 5:**
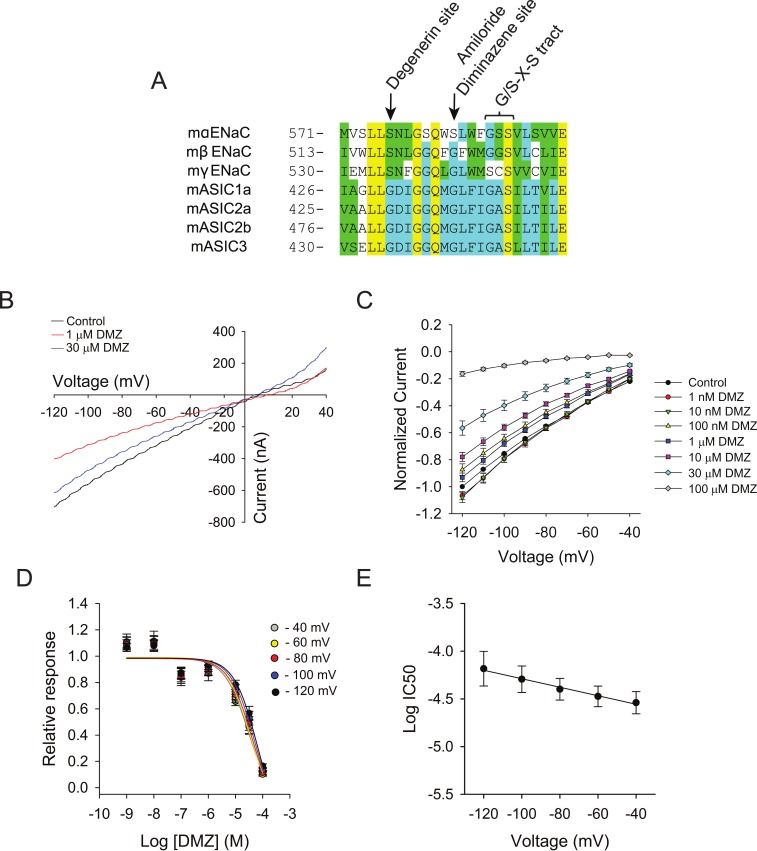
Diminazene binds to the pore of ASIC1a. (A) Sequence alignment for residues in the pore of ENaC/Deg channels. Note that many of the pore lining residues are conserved among ENaC and ASIC subunits, including the putative amiloride binding site (Gly438 in ASIC1a). (B) Representative ramp tracings from an oocyte expressing the G438A mutant before and after diminazene. Ramps from +60 to -140 mV with a duration of 500 msec were generated in the absence and presence of diminazene (1 μM and 30 μM). ASIC currents were evoked by changing the extracellular pH from 8 to 6.5. (C) Normalized proton-evoked currents from oocytes expressing G438A channels in the absence and presence of diminazene. Proton-evoked currents measured in the presence of diminazene were normalized to the proton-evoked current measured at -120 mV in the absence of diminazene (n = 9–27). (D) Diminazene IC_50_ at each voltage was calculated from dose-response curves. The calculated diminazene IC_50_ for the G438A mutant was 29 μM (CI 16 to 55 μM, n = 9–27) at—40 mV, 34 μM (CI 20 to 60 μM, n = 9–27) at—60 mV, 40 μM (CI 24 to 74 μM, n = 9–27) at -80 mV, 51 μM (CI 30 to 98 μM, n = 9–27) at -100 mV, and 66 μM (CI 38 to 140 μM, n = 9–27) at -120 mV. (E) Ala mutation at position 438 abolishes voltage dependence of diminazene block. Log IC_50_ values (C) were plotted as a function of the membrane potential. Data represent log IC_50_ ± SEM. Data were fitted by linear regression (slope -0.0045 ± 0.0003, r^2^ = 0.987, p<0.001).

Amiloride is a potassium sparing diuretic that binds with high affinity to the pore of ENaC, and it is used clinically to treat hypertension and swelling due to heart failure or cirrhosis. Although amiloride and diminazene are not structurally related, we posited that they share a common mechanism of action based on the observation that amiloride’s inhibition of ENaC/Deg channels is affected by the membrane potential. ENaCs are heteromers made of three homologous subunits termed alpha, beta and gamma. Site-directed mutagenesis studies showed that residues alphaSer583, betaGly525, and gammaGly537 in the second transmembrane domain are part of the amiloride binding site on ENaC [44–46]. Many of the pore lining residues are conserved among ENaC and ASIC subunits, including the putative amiloride binding site, Gly438 in ASIC1a (Fig 5A). We posited that if diminazene binds to the pore of channel at a site homologous to the amiloride binding site in ENaC, then mutations at position Gly438 should affect diminazene binding and alter the voltage dependence of inhibition. To test this, we generated dose-response curves with channels bearing a Gly to Ala mutation at position 438. As shown in Fig 5B, currents increased linearly with the membrane potential in the presence of diminazene (compare with Fig 4A). Fig 5C shows normalized proton-evoked currents between membrane potentials of -40 mV and -120 mV. I-V relationships were calculated by subtracting the currents measured at pH 8.0 from those measured at pH 6.5, as indicated above. Significantly, the block of the G438A mutant by diminazene did not show a dependence with the membrane potential (Fig 5D and 5E).

## Discussion

In this study we examined the molecular mechanisms of inhibition of ASIC1a by diminazene. We have identified two sites, one in the lower palm domain and another in the pore, where mutations alter diminazene block. Mutations of residues Glu79 and Glu416 in the palm domain reduced diminazene IC_50_ by 6–8 fold, while a single mutation in the pore at position 438 reduced diminazene IC_50_ by at least 10 fold. Below we discuss the significance of our findings and enunciate a mechanism for the inhibition of ASIC1a by diminazene.

A characteristic of all pore blockers is their dependence on membrane potential. Amiloride, a prototypic pore blocker of ENaC/Deg channels, binds to a site within the electric field of the membrane [47]. Consistent with the notion that diminazene binds to the pore of ASIC1a, the block of this channel by diminazene displayed a strong dependence with the membrane potential. In good agreement with our results, Wiemuth and Gründer reported that the inhibition of the brain liver Na^+^ channel (BLINaC) and ASIC1a by diminazene are affected by membrane potential [48, 49]. Mutagenesis studies have identified residues in the pore of the ENaC (alphaS583, betaG525 and gammaG537) that are critical for block by amiloride [44–46]. Moreover, analysis of cASIC1 crystals soaked in amiloride revealed the presence of three amiloride molecules partially occluding the extracellular vestibule of the pore [50]. In the soaked crystals, amiloride molecules are associated to residues Asp433 and Gln437 (corresponding to Asp432 and Gln436 in ASIC1a) through hydrogen bonds and van der Waals interactions, respectively. Because amiloride molecules are not occluding the ion channel pore, it was proposed that this site represents a binding site for amiloride as it enters the pore [50]. Our results show that a single Ala mutation at position 438 in the second transmembrane domain of ASIC1a, at a site homologous to the amiloride binding site on ENaC, reduced diminazene apparent affinity by one order of magnitude. We speculate that diminazene, similar to amiloride [51], binds in the vicinity of Gly438, occluding the ion conduction pathway. This result is in good agreement with the work of Schmidt and colleagues, which showed that single Cys mutations at positions Gly435, Gln436, Leu439, Phe440 and Ile441 in the pore of ASIC1a reduce diminazene affinity [48]. Note that mutations can alter the structure of the pore, hence it is not possible to define the precise binding site for diminazene using site-directed mutagenesis. Significantly, our studies showed that the G438A mutation abolishes the voltage dependence of diminazene block. This finding suggests that this mutation prevents the binding of diminazene to the pore of ASIC1a and supports the notion that diminazene binds also to a site outside the pore of the channel (see below).

We previously described the presence of two proton coordination sites in the extracellular region of ASIC1a, one in the lower palm domain formed by residues Glu79 and Glu416, and another sensitive to the covalent modification of residues in the finger-thumb interface [41, 42]. These two proton sensors jointly facilitate pore opening in response to extracellular acidification [42]. Significantly, individual neutralization of Glu79 and Glu416 in the lower palm domain results in shifted proton activation curves [42]. Moreover, using thiol reactive reagents we showed that residues at position 79 and 416 are accessible for covalent modification when ASIC1a resides in the closed state, but they are buried inside the protein when the channel resides in the desensitized state [41]. Together, these findings suggested that upon protonation of residues Glu79 and Glu416, the lower palm domain undergoes a conformational change that triggers pore opening [41, 42]. Because diminazene is charged at physiological pHs, we posited that it might bind to this array of acidic residues in the lower palm domain. Our mutagenesis studies revealed that the introduction of neutral residues at positions 79 and 416 in ASIC1a reduce diminazene IC_50_ by 6–7 fold. In contrast, neutralizing mutations of residues in the acidic pocket of ASIC1a did not alter diminazene inhibition. We propose that the interaction of diminazene with these residues restricts conformational changes associated with pore opening, reducing the magnitude of the response to extracellular acidification. Because Gln mutation at positions 79 and 416 accelerate desensitization [41], we were not able to assess the voltage dependence of diminazene block in these mutants.

In summary, our studies have identified two regions in ASIC1a where mutations alter diminazene block, the lower palm domain and the pore. These results indicate that the inhibition of ASIC1a by diminazene involves both allosteric modulation and blocking of ion flow through the conduction pathway. To the best of our knowledge, diminazene is the first small molecule identified that targets the pore of ASICs with high affinity. Because diminazene and related diarylamidines (DAPI, hydroxysitlbamidine or pentamidine) do not inhibit ENaC activity [38], these molecules could perhaps be modified to improve potency while maintaining specificity toward ASICs.
